# The double-edged sword of probiotic supplementation on gut microbiota structure in *Helicobacter pylori* management

**DOI:** 10.1080/19490976.2022.2108655

**Published:** 2022-08-11

**Authors:** Ali Nabavi-Rad, Amir Sadeghi, Hamid Asadzadeh Aghdaei, Abbas Yadegar, Sinéad Marian Smith, Mohammad Reza Zali

**Affiliations:** a Foodborne and Waterborne Diseases Research Center, Research Institute for Gastroenterology and Liver Diseases, Shahid Beheshti University of Medical Sciences, Tehran, Iran; b Gastroenterology and Liver Diseases Research Center, Research Institute for Gastroenterology and Liver Diseases, Shahid Beheshti University of Medical Sciences, Tehran, Iran; c Basic and Molecular Epidemiology of Gastrointestinal Disorders Research Center, Research Institute for Gastroenterology and Liver Diseases, Shahid Beheshti University of Medical Sciences, Tehran, Iran; d Department of Clinical Medicine, School of Medicine, Trinity College Dublin, Dublin, Ireland

**Keywords:** Helicobacter pylori, gastrointestinal microbiota, gut metabolome, probiotic supplementation, intestinal homeostasis, metabolic disorder, gastric cancer

## Abstract

As *Helicobacter pylori* management has become more challenging and less efficient over the last decade, the interest in innovative interventions is growing by the day. Probiotic co-supplementation to antibiotic therapies is reported in several studies, presenting a moderate reduction in drug-related side effects and a promotion in positive treatment outcomes. However, the significance of gut microbiota involvement in the competence of probiotic co-supplementation is emphasized by a few researchers, indicating the alteration in the host gastrointestinal microbiota following probiotic and drug uptake. Due to the lack of long-term follow-up studies to determine the efficiency of probiotic intervention in *H. pylori* eradication, and the delicate interaction of the gut microbiota with the host wellness, this review aims to discuss the gut microbiota alteration by probiotic co-supplementation in *H. pylori* management to predict the comprehensive effectiveness of probiotic oral administration.

**Abbreviations**: acyl-CoA- acyl-coenzyme A; AMP- antimicrobial peptide; AMPK- AMP-activated protein kinase; AP-1- activator protein 1; BA- bile acid; BAR- bile acid receptor; BCAA- branched-chain amino acid; C2- acetate; C3- propionate; C4- butyrate; C5- valeric acid; CagA- Cytotoxin-associated gene A; cAMP- cyclic adenosine monophosphate; CD- Crohn’s disease; CDI- C. difficile infection; COX-2- cyclooxygenase-2; DC- dendritic cell; EMT- epithelial-mesenchymal transition; FMO- flavin monooxygenases; FXR- farnesoid X receptor; GPBAR1- G-protein-coupled bile acid receptor 1; GPR4- G protein-coupled receptor 4; H2O2- hydrogen peroxide; HCC- hepatocellular carcinoma; HSC- hepatic stellate cell; IBD- inflammatory bowel disease; IBS- irritable bowel syndrome; IFN-γ- interferon-gamma; IgA immunoglobulin A; IL- interleukin; iNOS- induced nitric oxide synthase; JAK1- janus kinase 1; JAM-A- junctional adhesion molecule A; LAB- lactic acid bacteria; LPS- lipopolysaccharide; MALT- mucosa-associated lymphoid tissue; MAMP- microbe-associated molecular pattern; MCP-1- monocyte chemoattractant protein-1; MDR- multiple drug resistance; mTOR- mammalian target of rapamycin; MUC- mucin; NAFLD- nonalcoholic fatty liver disease; NF-κB- nuclear factor kappa B; NK- natural killer; NLRP3- NLR family pyrin domain containing 3; NOC- N-nitroso compounds; NOD- nucleotide-binding oligomerization domain; PICRUSt- phylogenetic investigation of communities by reconstruction of unobserved states; PRR- pattern recognition receptor; RA- retinoic acid; RNS- reactive nitrogen species; ROS- reactive oxygen species; rRNA- ribosomal RNA; SCFA- short-chain fatty acids; SDR- single drug resistance; SIgA- secretory immunoglobulin A; STAT3- signal transducer and activator of transcription 3; T1D- type 1 diabetes; T2D- type 2 diabetes; Th17- T helper 17; TLR- toll-like receptor; TMAO- trimethylamine N-oxide; TML- trimethyllysine; TNF-α- tumor necrosis factor-alpha; Tr1- type 1 regulatory T cell; Treg- regulatory T cell; UC- ulcerative colitis; VacA- Vacuolating toxin A.

## Introduction

Gastric carcinoma, as one of the leading causes of cancer-associated deaths, is mainly developed as a result of *Helicobacter pylori* (*H. pylori*) infection. The prevalence of *H. pylori* infection exceeds half of the world’s population; however, the likelihood of affecting health or disease is not uniform and largely relies on host genetics, bacterial virulence, and environmental conditions.^1^ By leveraging several virulence factors, *H. pylori* interferes with various cellular components of the host to induce proliferation, apoptosis, migration, and inflammatory responses.^2^ *H. pylori* has a substantial association with chronic gastritis, gastric ulcer, mucosa-associated lymphoid tissue (MALT) lymphoma, and gastric adenocarcinoma.^3^ The combination of up to four drugs including two or three types of antibiotics as well as a proton-pump inhibitor for two weeks is suggested as the first-line of *H. pylori* treatment.^4–6^ However, the ideal approach for *H. pylori* eradication remains elusive and current prescriptions are mostly empirical, heedless of the bacterial antibiotic susceptibility.^7^

The increased prevalence of antibiotic resistance and antibiotic-associated adverse effects are the primary reasons explaining the requirement for alternative approaches to manage *H. pylori* infection.^8^ The interaction of probiotics with the host and gastrointestinal microbiome through alteration in the gut microbiota composition, competition for accessible nutrients and attachment sites, and prevention of bacterial colonization to mediate health benefits indicates the advantage of probiotic co-supplementation in antibiotic treatments.^9^ Intervention studies have demonstrated a reduction in gastrointestinal symptoms and drug-related side effects by probiotic oral administration.^10^ However, long-term follow-up investigations are required to elucidate the efficiency of adjuvant interventions on *H. pylori* treatment.

Here, we aim to highlight the great significance of the host gut microbiota involvement in the competence of probiotic supplementation. We will further discuss the bidirectional interaction of probiotic strains and indigenous gastrointestinal microbiota to predict the effectiveness of this adjuvant therapy and provide an outlook for future investigations within the nascent and promising research field.

## Gut microbiota

**Figure 1.**
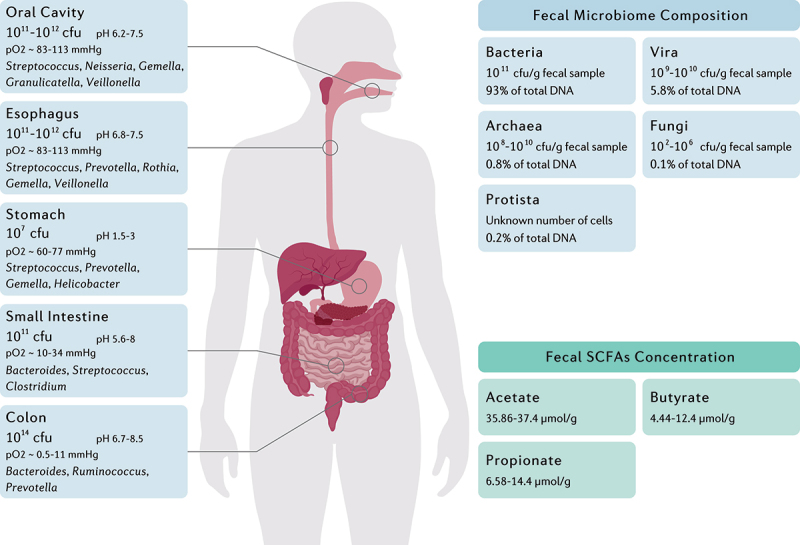
The main genera and total abundance of bacteria vary along the gastrointestinal tract. The colon is characterized by low levels of oxygen as well as the presence of enormous numbers and species of bacteria. On the other hand, the microbial composition and metabolite concentration of stool samples are distinguished from gut biopsies, in which the bacteria and the fungi constitute the majority and minority of total fecal DNA, respectively.^12–15^ Fecal concentration of SCFAs are also demonstrated as they might be considered key regulators of the intestinal homeostasis.

In addition to the tremendous community of microorganisms inside and on the human body, the gastrointestinal tract harbors a diverse and dynamic consortia of commensal or mutualistic microorganisms, mainly consisting of Firmicutes, Bacteroidetes, Actinobacteria, Proteobacteria, and Verrucomicrobia phyla.^11^ Based on the ecological characteristics of the gastrointestinal tract, the microbial load ranges from 10^12^ CFU/ml in the oral cavity and a narrow diversity of 10^7^ CFU/ml in the stomach and duodenum to a vast diversity of 10^14^ CFU/ml in the colon (Figure 1).^12^ Due to the reduction in oxygen concentration along the longitudinal axis, the upper gastrointestinal tract is the residence of Gram-positive cocci, such as *Gemella* and *Streptococcus*, whereas the intestines and colon are enriched with anaerobes including the *Clostridium* and *Faecalibacterium* genera.^16^ Furthermore, the luminal to mucosal axis organizes the bacteria based on their ability for mucus degradation. *Bacteroides thetaiotaomicron*, *Akkermansia muciniphila*, *Ruminoccous gnavus*, *Bacteroides fragilis*, and *Bifidobacterium bifidium* are predominant bacteria within the mucus layer that utilize glycans as their energy source by glycosidase, sulphatase, and sialidase enzymes.^17^ Despite the dynamic colonization of indigenous commensals within the intestinal niches created by glycans, the lack of dietary fiber polysaccharides potentially emphasizes the significance of the host intestinal mucin as a reliable energy source for the gut microbiota.^18^

### The role of immune system in shaping gut microbiota

A distinctive characteristic of the intestinal immune system is its capacity to distinguish mutualistic microorganisms from pathogens and further establish active tolerance toward commensal bacteria.^19^ Identification of microbe-associated molecular patterns (MAMPs) by pattern recognition receptors (PRRs), such as toll-like receptors (TLRs) and nucleotide-binding oligomerization domain receptors (NODs), leads to the activation of various cellular signaling pathways. Consequently, modulation of gene expression by multiple ligands, transcription factors, and kinases can modify the production levels of inflammatory cytokines, chemokines, and immunoreceptors.^20^ Although pathogens and commensals share common ligands that activate the TLRs, several mechanisms are considered for TLR-mediated discrimination of gut bacteria. Commensals can be simply distinguished from pathogens owing to the lack of virulence factors and differences in invasiveness. Furthermore, the cellular location of TLRs on the intestinal epithelium is inaccessible to commensal bacteria. Different PAMP affinity for TLRs and activation of ligand-specific signaling pathways are other possible mechanisms to identify commensals from pathogens.^21^ On the other hand, NOD2 recognizes conserved motifs of bacterial peptidoglycan and maintains mucus layer activity; thereby, NOD2 deficiency or mutation might lead to pathogen overgrowth, inflammation, and colon cancer.^22^ A recent study indicated that NOD2 knockout mice demonstrated an impaired recovery of gut microbiota composition following an antibiotic intervention, suggesting the remarkable contribution of this receptor in shaping the gut microbial community.^23^ Furthermore, NOD1 activation as a consequence of peptidoglycan recognition can trigger both immune memory and tolerance.^24^ Irving et al. demonstrated the development of peptidoglycan-specific immunity following *H. pylori* infection and the subsequent NOD1 activation and autophagy induction.^25^

The mucus layer of the intestinal epithelium intervenes between the resident microbiome and epithelial layer to form a static shield and narrow the immunogenicity of antigens by provoking dendritic cells (DCs) to an anti-inflammatory response. Moreover, the complex architecture of the intestinal epithelium, as well as their secretions, such as antimicrobial peptides (AMPs) and immunoglobulins, preserve the functionality of the mucosal barrier.^26^ The most abundant AMPs are defensins that develop small pores in bacterial membranes to disrupt cellular integrity. α- and β-defensins are the two subfamilies of defensins, predominantly released by Paneth cells and colonic epithelial cells, respectively.^27^ In addition to pore formation, these AMPs can trap bacteria by degenerating the bacterial cytoplasm and developing extracellular net-like structures.^28^ Furthermore, cathelicidin is the primary AMP expressed during infancy regardless of the bacterial presence and remarkably influences the early development of gut microbiota.^29^ Perturbation of the gastrointestinal microbiota of preterm and term infants may lead to persistent immune and metabolic disorders.^30^ Collectively, the intestinal epithelium can establish an efficacious physico-chemical barrier that prevents pathogen colonization on the mucosal surface while creating immune tolerance against commensal bacteria.

In addition to the innate immune system, recent studies exhibited a mutualistic interaction of the adaptive immune system in shaping gut microbial composition. B cells are critical modulators of intestinal homeostasis, mainly through expressing secretory immunoglobulin A (SIgA) in response to commensal recognition.^31^ The pivotal and often oversimplified role of SIgA depends on the gut microbial community. Chaotic or excessive reaction to alteration in the richness or pro-inflammatory behavior of particular strains by SIgA influences not only the specific bacteria but probably the whole microbiota.^32^ SIgA predominantly prevents the translocation of microorganisms from lamina propria to the bloodstream, interferes with conjugative plasmid transfer, and facilitates the colonization of commensal bacteria.^33^ On the other hand, T follicular helper cells are specialized to cooperate with B cells and modify humoral immunity.^34^ Although several studies began to elucidate the mechanistic interaction of cellular immunity with gut microbiota through inflammatory signaling pathways, we have yet to fully understand the aspects of the adaptive immune system in shaping the gut microbiota.

### Gut microbial metabolites in preserving homeostasis

The gut microbiota plays a critical role in preserving the normal bioactivity of the host through gut microbiota-derived metabolites, especially bile acids (BAs), short-chain fatty acids (SCFAs), branched-chain amino acids (BCAAs), trimethylamine N-oxide (TMAO), tryptophan, and indole derivatives.^35^ Nevertheless, the knowledge concerning the direct effect of the gut microbiota on the host metabolism remains scarce; however, the gastrointestinal microbiota has a particular interaction with mitochondria owing to their common origin.^36^ It has been recently indicated that delta-valerobetaine production by the gut microbiome reduces cellular carnitine and mitochondrial long-chain acyl-coenzyme A (acyl-CoA); consequently, this obesogenic metabolite prevents mitochondrial fatty acid oxidation and leads to diet-dependent obesity.^37^

**Figure 2.**
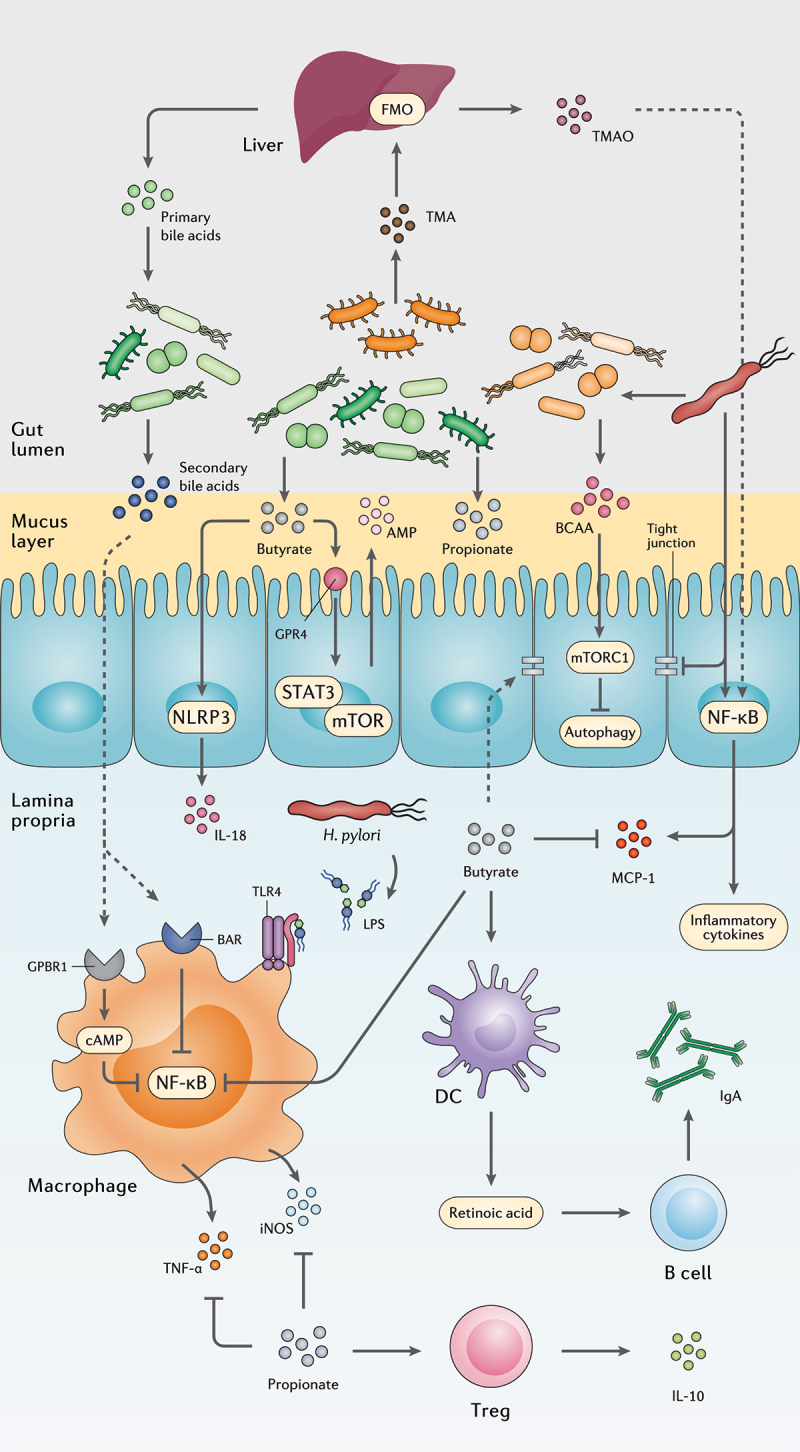
The interplay between the gut metabolome, *H. pylori*, and the host immune system. *H. pylori* induces chronic gastric inflammation through the activation of transcriptional factors such as NF-κB. By stimulating the production of BCAA from the gut microbiota, *H. pylori* activates the mTORC1 complex and ultimately inhibits autophagic response. *H. pylori* further disrupts the integrity of the gastric epithelial barrier by suppressing the expression of tight junction proteins. On the other hand, microbiota production of SCFAs and secondary bile acids modulate gastric inflammation and immune system activation by reducing NF-κB activation, promoting the secretion of anti-inflammatory cytokines, AMPs, and IgA, and preserving the integrity of the gut barrier.

SCFAs are saturated fatty acids acquired from microbiota-accessible carbohydrates and mainly include acetate (C2), propionate (C3), butyrate (C4), and valeric acid (C5) in the human body.^38,39^ Nevertheless, the abundance of each SCFA depends on substrate availability, gut microbiota composition, and gastrointestinal transit time. SCFAs exhibit several local effects, such as preserving the intestinal barrier integrity and pH reduction as their concentration increase from the distal ileum (6.5–7.5) to the proximal colon (5.5–7.5).^40,41^ Moreover, SCFAs promote the induction and expansion of intestinal regulatory T cells,^42^ DCs, and macrophages,^43^ exert an anti-carcinogenic and anti-oxidative effect in the intestine,^44^ and suppress pathogen-induced inflammation (Figure 2).^45^

Hepatocytes synthesize primary bile acids from cholesterol, conjugate them to taurine or glycine, and then release them into the gall bladder to form bile in combination with cholesterol, phospholipids, minerals, electrolytes, bilirubin, biliverdin, and protein.^46^ Intestinal bacteria will deconjugate primary BAs that fail reabsorption in the terminal ileum and thereby convert them to secondary BAs by microbial biotransformation, including dehydroxylation, epimerization, and oxidation of hydroxyl groups.^47^ Secondary BAs are involved in the modulation of cell signaling, microbial composition, intestinal metabolism, and the host immune response. Reduced BA deconjugation is associated with inflammatory bowel diseases (IBD) including ulcerative colitis (UC) and Crohn’s disease (CD), as well as irritable bowel syndrome (IBS).^48^ Free BAs, such as cholic acid, deoxycholic acid, and chenodeoxycholic acid, can stimulate apoptosis and reduce interleukin 6 (IL-6) production, while conjugated BAs such as glycolic acid, glycodeoxycholic acid, and glycochenodeoxycholic acid promote cell growth and induce IL-6 production.^49^ However, excessive production of the secondary BA deoxycholic acid triggers the expression of inflammatory and tumorigenic factors in hepatic stellate cells (HSCs), contributing to hepatocellular carcinoma development.^50^ Secondary BAs might also activate farnesoid X receptor (FXR) and elevate the risk of developing colorectal cancer and hepatocellular carcinoma.^51^

As an essential amino acid in the human body, tryptophan must be obtained by diet and further metabolized through host or microbial pathways. The indole pathway for tryptophan metabolism is mediated by the gut microbiome leading to a variety of indole metabolites, some of which are involved in mucosal homeostasis, gastrointestinal motility, and the host immune response.^52^ However, BCAAs valine, leucine, and isoleucine are possible biomarkers in human carcinogens owing to their requirement in cancer cell growth and tumor progression.^53^ Although BCAAs are involved in carcinogenesis and metabolic disorders, such as obesity, insulin resistance, and type 2 diabetes (T2D), sports supplements with these amino acids might improve strenuous training.^54^

Gut bacteria produces TMA, which is transferred to the liver through the bloodstream and further converted to TMAO by hepatic flavin monooxygenases (FMOs). Animal products such as meat, fish, and eggs are rich in TMA precursors.^55^ TMAO is a major risk factor for cardiovascular disease, renal fibrosis and functional impairment, atherosclerosis, and colorectal cancer.^56,57^ It is further indicated that a precursor to TMAO, trimethyllysine (TML), alone and combined with TAMO, is involved in cardiovascular events for patients with the acute coronary syndrome.^58^

### Gut microbial dysbiosis

Defining the gut microbiota composition and function of metabolically healthy individuals is a prerequisite for claiming gut dysbiosis and identifying disease-related biomarkers. The efforts in this field are encountered with an intimidating complexity in the host–microbiota interaction, which needs comprehensive, multidisciplinary approaches for further elucidation.^59^ Although a healthy microbiome composition is yet to be determined, the relative alteration of gastrointestinal microorganisms in disease conditions can be mainly classified as pathobiont enrichment, commensal depletion, or diversity reduction.^60^ Pathobionts are among the host indigenous microbiome that can trigger or accelerate diseases in particular genetic or environmental conditions.^61^ An increased proportion of Enterobacteriaceae, including *Escherichia coli, Klebsiella* spp., and *Proteus* spp., is a typical example of pathobionts enrichment. This family of Gram-negative symbionts is commonly overgrown in multiple inflammatory situations including intestinal bowel disease, obesity, celiac disease, colon cancer, and antibiotic therapies.^62^ In contrast to the overgrowth of pathobionts, the gut microbial community frequently suffers a tremendous depletion or total loss of some commensal bacteria following microbial elimination or reduced bacterial proliferation.^60^ Commensal bacteria are responsible for providing energy resources for the host enterocytes,^63^ inhibiting pathogen colonization,^64^ preserving lymphoid tissue architecture, and regulating the immune response.^26^ Bio-engineered commensal supplementation is an innovative strategy, recently used for delivering tailored substances to target particular metabolic pathways.^65^ On the other hand, a common and recurrent feature of disease-related dysbiosis is reduced microbial diversity. Although reduced alpha diversity might be the effect rather than the cause of disorders, this characteristic is correlated to gastrointestinal and extra-gastrointestinal diseases, such as CD, IBS, colorectal cancer, and autism.^66^ Furthermore, the development of a mature microbiome through lifespan highly relies on alpha diversity. Interestingly, specific bacteria can be used as markers for the development and maturation of the microbiota such as *R. gnavus*, which is inversely correlated to microbial richness at all ages and reduces from childhood toward adulthood.^67^ Accordingly, there is a delicate interaction between gut homeostasis and the host biological function. Disruption of the intricate equilibrium of metabolic interactions by pathogen colonization or microbiota modifying interventions can damage the integrity of the gut barrier, change the host indigenous bacteria, and further lead to metabolic disorders.

## *H. pylori* and gut microbiota

**Figure 3.**
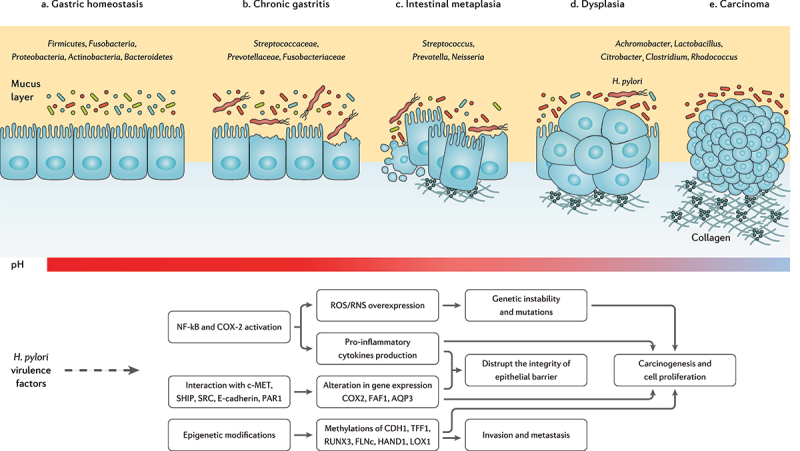
The progression of chronic gastritis toward gastric carcinoma has been characterized by the reduction in the *Helicobacter* genus, overgrowth of opportunistic bacteria, increased apoptosis, necrosis, and collagen production, changes in the cytoskeleton and polarity of the gastric epithelium, and gradual suppression of gastric acidity. The main mechanisms of action through which *H. pylori* virulence factors promote the risk of developing gastric cancer are further depicted.^71–73^

The clinical implications of *H. pylori* infection are not limited to gastrointestinal disorders but also include *H. pylori* association with obesity, diabetes, IBD, allergic disorders, as well as cardiovascular, hepatobiliary, skin, kidney, autoimmune, neurologic, and psychiatric diseases.^68^ This might indicate the importance of *H. pylori* and gut microbiota crosstalk, as several mechanisms are reported for this pathogen influencing the host microbiome.^69^ Modulation of the host immune response, manipulation of the cellular signaling, impairment of the epithelial cell polarity, and alteration of gastric acidity are the primary mechanisms contributing to gut microbiota alteration during *H. pylori* infection.^70^ Below, we discuss several aspects of *H. pylori* infection interacting with gastric and intestinal microbiome, as well as gut microbial metabolites (Figure 3).

### Gastric microbiota

In the last decade, several studies have compared the gut microbiota composition of *H. pylori*-infected and non-infected individuals, reporting controversial data even regarding the diversity and richness of the microbial community.^74^ It is possibly due to the remarkable dependence of microbiota composition on individual and environmental factors, such as host genetics, ethnicity, geography, socioeconomic status, and diet.^75,76^ Furthermore, the microbial community is highly variable along the longitudinal axis of gastrointestinal tract. Hence, in a recent study, corpus and antrum bacteria were reported to significantly differ between individuals positive or negative for *H. pylori*, while the bacterial community from the lower gastrointestinal tract and stool samples were comparable.^77^

Although *H. pylori* antigen load exhibited a reverse relationship with *Fusicatenibacter, Alistipes, Bacteroides*, and *Barnesiella* genera, gut microbiota composition is mainly dominated by the same phyla yet different richness in *H. pylori*-infected and non-infected individuals.^72^ *Streptococcus, Neisseriae, Prevotella, Rothia, Fusobacterium, Veillonella*, and *Haemophilus* are considered the main gastric bacterial genera enriched in *H. pylori*-positive individuals, compared to *H. pylori*-negative subjects.^78,79^ Likewise, the overgrowth of *Candida* species in the stomach has been reported during *H. pylori* infection, which might result in synergistic effects on the *H. pylori* pathogenesis.^80^ However, *H. pylori*-induced gastric microbiota alteration is strain-specific and independent of the host-microbial colonization burden. A recent in vivo study demonstrated the substantial reduction of *Akkermansia, Bacteroides*, and *Lachnospiraceae* genera in gerbils infected with a cytotoxin-associated gene A (CagA)-positive *H. pylori* strain compared to a CagA-negative strain. Yet, comparable alpha diversity for the gastric microbiota has been reported for the investigated groups.^81^ Furthermore, allelic variation in the *H. pylori* vacuolating toxin A (VacA) is associated with distinct modification of the gastric microbiota.^82^

The microbiome alteration further relies on the stages of gastric tumorigenesis along with substantial enrichment of oral microbial species including *Peptostreptococcus stomatis, Streptococcus anginosus, Parvimonas micra, Slackia exigua*, and *Dialister pneumosintes* toward carcinogenesis.^83^ Some studies reported an increased colonization burden and microbial diversity, as well as the overgrowth of cancer promoting bacteria in the gastric mucosa of patients with gastric cancer compared to gastritis.^84,85^ However, a metagenomics study indicated that the microbiota tends to be gradually depleted in the gastric mucosa from non-atrophic gastritis toward intestinal metaplasia and gastric cancer. In this regard, a significant reduction in TM7, *Porphyromonas* sp, *Neisseria* sp, and *Streptococcus sinensis*, as well as a substantial enrichment in *Lactobacillus coleohominis* and Lachnospiraceae have been further reported.^86^

Even though *Helicobacter* is the most abundant genus in chronic gastritis, gastric carcinoma is reported with a significant reduction in the proportion of this genus. Meanwhile, certain commensals but potentially opportunistic pathogenic taxa such as *Citrobacter, Clostridium, Lactobacillus, Achromobacter*, and *Rhodococcus* were found to be enriched among gastric microbiota in gastric cancer.^87^ Another study further reported *Streptococcus, Lactobacillus, Veillonella, Prevotella, Neisseria*, and *Haemophilus* as the highly prevalent gastric microbial genera in patients with gastric carcinoma.^88^ Consistent with the foregoing data, an enriched proportion of *Fusobacterium, Neisseria, Prevotella, Veillonella*, and *Rothia* genera have been characterized in patients with advanced gastric lesion compared to the healthy/superficial gastritis group.^89^

Lactic acid bacteria are mainly reported as protective bacteria in gastric carcinoma, while their increased abundance during cancer progression might indicate otherwise. Reactive oxygen species (ROS), N-nitroso compounds (NOC), and lactate production, as well as induction of epithelial–mesenchymal transition (EMT) and immune tolerance, are among carcinogenic factors promoted by lactic acid bacteria.^90^ On the other hand, the destruction of stomach hydrochloric acid-producing glands by *H. pylori* infection increases the stomach pH and eventually promotes the colonization of NOC-producing bacteria.^91,92^ *Veillonella, Clostridium, Haemophilus, Staphylococcus, Neisseria, Lactobacillus*, and *Nitrospirae* are involved in gastric carcinogenesis by NOC production and further induction of mutagenesis, angiogenesis, and proto-oncogenes expression as well as apoptosis prevention.^90^

### Intestinal microbiota

Compared to studies exploring the influence of *H. pylori* on the gastric microbiota, a limited number of studies investigated the effect of *H. pylori* on the intestinal microbiota. Considering the intestinal microbiota at the phylum level, Firmicutes, Proteobacteria, Actinobacteria, and Acidobacteria have been elevated, while Bacteroidetes has been reduced following *H. pylori* infection.^93,94^ At the genus level, *Bacteroides, Barnesiella, Alistipes*, and *Fusicatenibacter* have been negatively associated with *H. pylori* stool antigen load.^95^ Lapidot et al. also demonstrated a strong association between *H. pylori* infection and *Prevotella copri* and *Eubacterium biforme* in school-age children.^96^ Additionally, long-term *H. pylori* infection of Mongolian gerbils has been characterized by *Akkermansia* enrichment in the colon.^97^ Moreover, *Candida glabrata* and other unclassified fungi have been reported to be increased in stool samples following *H. pylori* infection in adults.^98^ However, regarding the alpha diversity of the intestinal microbiota, contradictory reports indicated microbial enrichment,^98–100^ microbial depletion^101^ or no significant alteration^102–104^ in *H. pylori*-infected patients. Except for one study, no significant alteration has been indicated for microbial alpha diversity following *H. pylori* infection. This might suggest that *H. pylori* promotes the host’s resilience against microbial depletion, reflecting the co-evolution of *H. pylori* and humans over tens of thousands of years.^95,105^ Furthermore, the geological and cultural differences among the investigated population might be responsible for the inconsistency in the aforementioned studies.^106^ Several aspects of *H. pylori*-induced alteration of intestinal microbiota remain to be further investigated. However, *H. pylori*-induced gastric immunopathogenesis including hypochlorhydria and hypergastrinemia is held responsible for *H. pylori*-associated intestinal dysbiosis.^107,108^

### Gut metabolome

*H. pylori* interactions with epithelial cells results in disruption of tight junctions and activation of the host inflammatory responses (Figure 2).^109^ This recalcitrant pathogen provokes the activity of the nuclear factor kappa B (NF-κB) transcription factor, stimulates the expression of monocyte chemoattractant protein-1 (MCP-1) from epithelial cells to induce monocyte infiltration, and activates monocytes through LPS interaction with TLR4. Consequently, *H. pylori* infection leads to the overexpression of pro-inflammatory cytokines including induced nitric oxide synthase (iNOS), tumor necrosis factor-α (TNF-α), interferon-gamma (IFN-γ), IL-8, IL-6, IL-4, and IL-1β.^110^

The interaction between *H. pylori* infection and SCFA is far from being fully elucidated, yet the reduction of SCFA has been reported in the feces of *H. pylori*-infected mice.^111^ Specifically, butyrate promotes intestinal barrier function via activating AMP-activated protein kinase (AMPK) or inhibiting claudin-2 production to stimulate the expression of tight junction proteins.^40^ Through the G protein-coupled receptor 4 (GPR4) and mammalian target of rapamycin (mTOR)/signal transducer and activator of transcription 3 (STAT3) signaling pathway, butyrate promotes AMPs expression in epithelial cells. SCFAs might lead to NLR family pyrin domain containing 3 (NLRP3) inflammasome activation by GPR4 receptor inducing IL-18 secretion from the epithelium. GPR109A is a surface receptor on DCs and macrophages that detects butyrate and further induces the development of regulatory T cells (Treg) and prevents the proliferation of T helper 17 (Th17) cells.^112^ Moreover, butyrate can suppress the production of iNOS, TNF-α, IL-6, MCP-1, and IFN-γ by inhibiting NF-κB activation.^113^ On the other hand, propionate downregulates the production of pro-inflammatory cytokines including IL-4, IL-5, and IL-17A, and stimulates Treg cells to release the anti-inflammatory cytokine IL-10. In LPS-activated monocytes, propionate is reported to inhibit TNF-α and iNOS expression.^45^ It is also suggested that the interaction of SCFAs with DCs elevates retinoic acid (RA) production and consequently increases IgA secretion by B cells in lamina propria.^114^

BAs interaction with bile acid receptor (BAR) in LPS-activated macrophages inhibits NF-κB transcription; therefore, downregulates the overexpression of pro-inflammatory cytokines. Furthermore, G-protein-coupled bile acid receptor 1 (GPBAR1) activation by BAs stimulates cyclic adenosine monophosphate (cAMP) production; therefore, BAs interfere with the NF-κB signaling pathway either directly or through competition of cAMP for the transcription region.^115^

VacA, as a major virulence factor in *H. pylori* bacteria, induces cellular autophagy to promote the growth and colonization of this pathogen in the mucosal layer.^116^ Thereafter, *H. pylori* may provoke the gut microbiome to produce BCAAs isoleucine, leucine, and valine, and thereby activates the mTORC1 complex to inhibit autophagy within the gut epithelium and further induces chronic inflammation.^117^ Another inflammatory metabolite in the intestine is TAMO, which induces NF-κB activation and promotes the expression of pro-inflammatory cytokines; consequently, a positive correlation has been reported between TAMO circular concentration and serum levels of IL-8 and TNF-α.^118,119^

## Microbiome modifying interventions

### Antibiotic therapy

Clinical studies have used innovative approaches including targeted sequencing of 16S ribosomal RNA (rRNA), PICRUSt (phylogenetic investigation of communities by reconstruction of unobserved states), and high-throughput DNA sequencing to facilitate the identification of microbial gene or taxon as disease biomarkers. Nonetheless, intra-individual variability of the gut microbiota, as well as microbiologically heterogeneous subjects has forced the host–microbiota interaction to remain fraught and challenging.^120^ Notwithstanding the individual distinctions in microbial composition and the enormous differences in pathologies of metabolic diseases, intervention in the fragile host-microbiota crosstalk can lead to joint and disease-specific alteration in the community and activity of gut microbiota. Obesity, T2D, cardio-metabolic disease, metabolic liver disease, and malnutrition are primary metabolic disorders resulting from microbiome dysbiosis.^121^

Antibiotic treatment as a major disrupter of the gastrointestinal microbial community may lead to alpha diversity reduction, metabolome alteration, and antibiotic resistance.^122^ Antibiotic administration not only influences the resistome of the subject to whom it is given, but also the whole population owing to selection for resistance to its function.^123^ The propagation and spread of antibiotic resistance genes in the mucus layer is a defensive function for gut microbiota to minimize the effect of antibiotics, yet short-term antibiotic therapy can cause a long-term reduction in certain commensal bacteria.^124^ In addition to the antibiotic-directed modification of the gut microbiota, researchers have reported that intervention therapies can remodel the gene expression and overall metabolic activity of the gastrointestinal microbiota.^125^ Moreover, PPIs as essential drugs in *H. pylori* eradication can directly disrupt microbial composition, in addition to increasing the stomach pH and thereby influencing which bacteria reach the intestine.^126^ It is also suggested that the gut microbiota response to antibiotic treatment is determined by particular bacteria in the pre-treatment microbiome; thereby, targeting these bacteria may reduce the risk of dysbiosis and antibiotic-related metabolic disorders.^127^

### Probiotic supplementation

Multiple microorganisms comply with the definition of probiotics as live microorganisms providing a health benefit when supplemented in sufficient amounts.^128^ The empirical top-down strategy to study indigenous bacteria enriched in healthy subjects is still a major approach to identify probiotic strains with sufficient beneficial effects on human health.^129^ Common probiotics classify as probiotic lactic acid bacteria (LAB) such as *Lactobacillus* spp., *Bifidobacterium* spp., and *Streptococcus* spp., non-LAB probiotics, such as *Clostridium butyricum, Bacillus* spp., and *E. coli* Nissle 1917, and next-generation probiotics, such as *Akkermansia muciniohila, Faecalibacterium prausnitzii*, and *Bacteroides* species.^130^

The impact of probiotic supplementation on human health has been largely investigated and reported to interfere with acute diarrhea, improve IBD, reduce the risk for late-onset neonatal sepsis, cardiometabolic syndrome, and necrotizing enterocolitis, increase *H. pylori* eradication rate, decrease the prevalence and intensity of respiratory infection, ease depression and manage atopic dermatitis.^131^ Although several studies have failed to investigate mucosal or fecal microbiota composition of individuals during therapeutic interventions, strong evidence points out that the effectiveness of probiotic strains might not rely on colonizing the gastrointestinal tract but rather reside in their capacity of sharing genes and metabolites, reinforcing disturbed bacteria, and directly affecting the gut barrier and immune cells.^132^ The differences in responding to the same probiotic supplementation in healthy adults further suggest that an individual’s basal gut microbiota influences the body’s response to probiotic strains.^133^ Considering the variabilities in the host genetics, diet, disease-associated dysbiosis, and indigenous gut microbiota composition, the responses to the same intervention therapy might differ within the study population.

### Next-generation probiotic supplementation

Next-generation probiotics, also termed as live biotherapeutics, emphasize emerging microorganisms not being used as health-promoting factors to date, which will probably be taken under a drug regulatory framework. Regarding the importance of the gut microbiota, these probiotic strains mainly originate from the human microbiome symbionts including *A. muciniphila, F. prausnitzii*, and several *Bacteroides* species.^134^ *A. muciniphila* as an abundant bacterium within the host intestine is involved in regulating metabolic pathways, modulating the immune response, and preserving the intestinal barrier.^135^ The prevalence of this bacterium is negatively associated with obesity, T2D, IBD, and appendicitis.^136^ Daily administration of 10^10^ *A. muciniphila* bacteria to obese volunteers for 90 days is reported to reduce insulin resistance, plasma cholesterol, and the risk for developing liver dysfunction and inflammation, whereas no significant alteration is demonstrated in the gut microbiota.^137^ On the other hand, *F. prausnitzii* is reported to be reduced in patients with IBD,^138^ IBS,^139^ colorectal cancer,^140^ obesity, and diabetes.^141^ Owing to the oxygen sensitivity of this bacterium and several other candidate strains, little is known about their efficiency and safety as probiotic supplements.^142^ It is suggested that prebiotic co-supplementation with next-generation probiotics may promote the survivability and activity of probiotic strains in the human gut.^143^ Nevertheless, the development of gastrointestinal modeling through organoid technology can deepen our knowledge of the complexity of probiotic-host interaction and provide the opportunity of designing personalized therapeutics and develop next-generation probiotics.^144^

## *H. pylori* eradication

International guidelines highly recommend *H. pylori* eradication for individuals who test positive.^145,146^ According to the test-and-treat strategy, randomized clinical trials were conducted to demonstrate the long-term safety of *H. pylori* treatment and further report that despite the transient alteration in gastrointestinal microbiota and elevation in specific antibiotic resistance, this perturbation diminished 8 weeks or one year after treatment. Meanwhile, the reduction in insulin resistance and triglyceride serum concentrations were demonstrated as the advantages of *H. pylori* management.^147^ Moreover, the incidence of developing gastric carcinogenesis can be decreased by 50% following therapeutic management of *H. pylori* infection.^148^ However, *H. pylori* eradication not only stimulates gut dysbiosis but may also selects out drug-resistant species from the gut microbiota and further expands single-drug resistance (SDR) and multiple-drug resistance (MDR) mechanisms in other microbial species.^149^ Furthermore, *H. pylori* eradication can lead to major drug-related side effects including T2D and gastric adenocarcinoma.^150,151^ The tight interaction of the gastrointestinal microbiota and host wellness, as well as microbiome alteration and alpha diversity reduction during intervention therapies suggest a substantial involvement of the host microbiota in the adverse effects of *H. pylori* treatment.^152^

As the gut microbiota can potentially spread the resistance genes from commensals to pathogens and regulate the host bioactivity,^54^ reducing antibiotic resistance genes and preserving the intrinsic gut microbiota composition might increase *H. pylori* eradication rate and reduce collateral damages. Probiotic supplementation during treatment can preserve the host indigenous microbiota, facilitate rebiosis, and restore the intrinsic balance of bacteria in the gastrointestinal tract.^153,154^ It has been recently indicated that probiotic administration reduces the resistome configuration in colonization-permissive individuals. However, post-treatment probiotic supplementation has been reported to inhibit the reduction of antibiotic resistance genes number and further spread the resistance mechanisms in the intestinal mucosa.^155^ Cifuentes et al. reported a substantial reduction in resistant genes for lincosamides, tetracyclines, MLS-B (macrolide, lincosamide, and streptogramin B), and beta-lactam class following *Saccharomyces boulardii* CNCM I-745 supplementation during *H. pylori* eradication.^156^ Moreover, a recent meta-analysis of 5792 cases indicated that probiotic supplementation significantly increases the *H. pylori* eradication rate. Zhang et al. further reported that long-term (>10 days) probiotic administration leads to a statistically higher eradication rate compared with short-term administration.^157^ However, limited effectiveness has been obtained in *H. pylori* eradication through probiotic supplementation as the main treatment strategy without being co-supplemented with conventional antibiotic regiments.^158^

Owing to the high prevalence of *H. pylori* infection in childhood, mostly adolescence, or young adulthood should be considered for screening studies.^148^ Clinical symptoms, epidemiology, diagnostic approaches, antibiotic susceptibility, and treatment strategies for *H. pylori* infection significantly differ from the ones in adults and children.^159^ Yet, significant improvement has been obtained in *H. pylori* management, decreasing clinical manifestations, and the incidence of antibiotic-related side effects through probiotic supplementation in children. *Lactobacillus casei* strains and multi-strain consortia of *Lactobacillus acidophilus* and *Lactobacillus rhamnosus* are reported as the foremost adjuvant supplement in promoting *H. pylori* eradication rate and reducing drug-associated adverse effects in children, respectively.^160^ However, major limitations to meta-analysis studies include the different study designs, the wide spectrum of the co-supplemented antibiotic regimen, and the few studies conducted on the same probiotic strain.^160^

## Probiotics mechanism of action in modulating *H. pylori* infection

**Figure 4.**
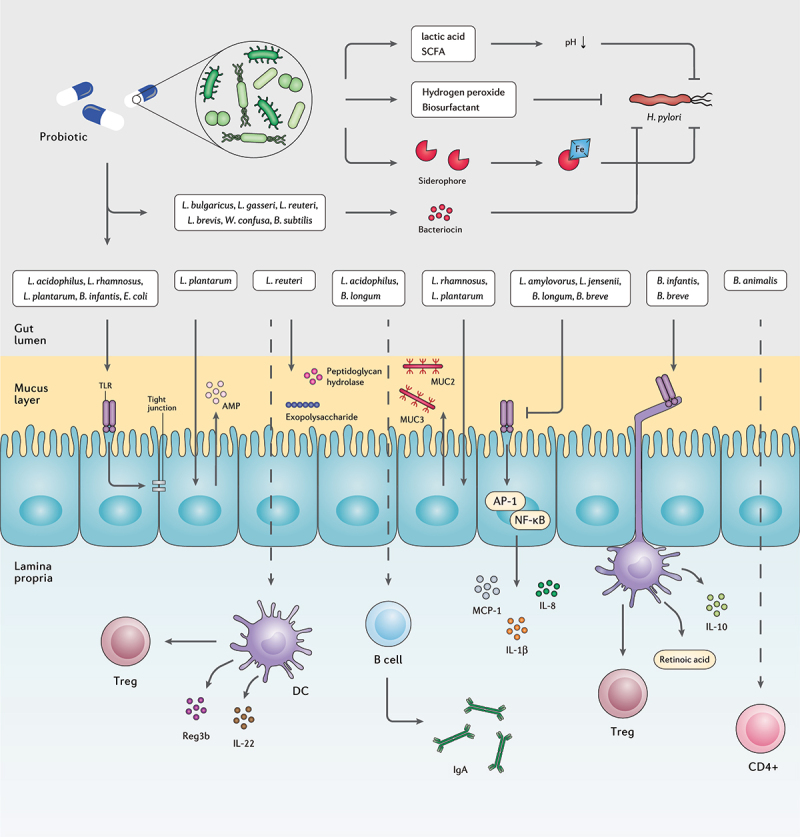
The interplay between probiotic strains, *H. pylori*, and the host immune system. Several probiotic strains can directly eliminate *H. pylori* cells by producing bacteriocins, siderophore, hydrogen peroxide, biosurfactant, lactic acid, and SCFAs. Probiotic bacteria can retain the activity of the gut barrier by stimulating the production of mucin and tight junction proteins. Certain probiotic species preserve the inherent structure of the gut microbiota by increasing the concentration of AMPs, peptidoglycan hydrolase, and exopolysaccharides. Furthermore, several probiotic bacteria regulate the host inflammatory response and prevent the development of chronic inflammation.

Studies have indicated that advantageous impacts of probiotics against *H. pylori* infection occur through a variety of mechanisms, such as reinforcement of gut mucosal barrier, elimination of pathogens, enhancement of the host immune system, and microbiome modification (Figure 4).^161^ Several probiotic species are antagonistic toward invasive pathogens, yet in *H. pylori* eradication, solid proof indicates that probiotics mainly reduce antibiotic-induced side effects.^162^ However, there are considerable limitations in these mechanistic studies including high reliability on cell-culture systems not attributed to complex intestinal environment and low colonization capacity of human probiotic strains in the gastrointestinal tract of mice models.^131^ Nonetheless, multiple key mechanisms are demonstrated for probiotic administration in clinical, in vitro, and in vivo studies, as detailed further below.

### Promotion of mucosal barrier

The gastrointestinal epithelium as the front line of the host innate defense against pathogenic invaders is required to preserve the integrity of the gastrointestinal barrier. Despite the uncovered mechanisms concerning the exact relationship between the intestinal barrier and inflammatory disorders, a defective epithelial barrier rather than immune dysfunction may result in chronic inflammation.^163^ Accordingly, *H. pylori*-associated carcinogenesis is either indirectly accelerated by chronic inflammation and tumorigenesis or directly through induction of epigenetic alteration in the gastric epithelial cells by bacterial factors.^164^

The protective properties of the mucosal barrier largely rely on the gut microbiota community and their components and metabolites. Due to the presence of mucin glycan, the so-called mucus-associated microorganisms can colonize and attach to the intestinal mucus layer.^165^ Recent advances in characterizing the beneficial mechanisms of commensal bacteria have led to novel strategies to maintain and promote intestinal barrier function. *Lactobacillus plantarum* ZS2058 as a probiotic can preserve the gut barrier function and permeability by modulating the expression of tight junctions and improving the intestinal epithelium.^166^ *L. plantarum* 299 v and *L. rhamnosus* GG promote the expression of key mucin genes mucin 2 (MUC2) and MUC3 to maintain the integrity of the intestinal barrier.^167^ Moreover, *L. plantarum* ZS2058 is reported enhancing the host defense peptides such as pBD2 and PG1-5; therefore, elevating the intestinal barrier function.^166^ As a key bacterium in healthcare-related gastrointestinal infection, *Clostridioides difficile* colonization in the intestine contributes to nosocomial diarrhea with significant morbidity and mortality.^168^ For which, *Lactobacillus reuteri* LMG P-27481 is demonstrated to provoke IL-10 production in immature DCs, repair the mucosal barrier function, and obtain a distinguish outcome in preventing *C. difficile* colonization and toxin load possibly by expressing bioactive molecules including exopolysaccharide and peptidoglycan hydrolases.^169^

*H. pylori* can overcome the epithelial barrier by mislocalizing or reducing the expression of tight junction transmembrane protein components including junctional adhesion molecule A (JAM-A) and further disrupt the tight junctional defense barrier.^170^ The aforementioned mechanism highlights the activity of probiotics, such as *L. rhamnosus* GG, *L. acidophilus, L. plantarum* MB452, *Bifidobacterium infantis* BB-02, and *E. coli* Nissle 1917 that stimulate TLR activation and further promote epithelial barrier by regulation of tight junction proteins production.^171^ Nevertheless, some strains such as *Lactobacillus amylovorus* DSM 16698 T and *Lactobacillus jensenii* TL2937 negatively regulate TLR activation to inhibit the expression of pro-inflammatory cytokines IL-8 and IL-1β. Moreover, *Bifidobacterium longum* BB536 and *Bifidobacterium breve* M-16 V can significantly suppress IL-8, IL-6, and MCP-1 secretion by inhibiting activator protein 1 (AP-1) and NF-κB activation through interaction with TLR and increasing the expression of ubiquitin editing protein A20.^172^

### Secretion of antimicrobial substances

Lactic acid, SCFAs, hydrogen peroxide, and bacteriocin are the major antibacterial substances secreted from probiotics. The incomplete ionization of lactic acid and SCFAs act as proton carriers, lowering the cytoplasmic pH and accumulating toxic anions to prevent *H. pylori* colonization. Probiotics can further eliminate *H. pylori* by generating hydrogen peroxide (H_2_O_2_) and damaging pathogenic proteins, membrane lipids, and DNA of the bacterial cell.^173^ However, due to their oxygen tolerance, lactic acid bacteria have anti-oxidative properties suppressing oxidative stress through radical scavenging, metal ion chelation, antioxidant enzyme expression, and host antioxidant and ROS-producing enzyme regulation.^174^

Bacteriocin expression has been considered as a pivotal property of probiotics to inhibit pathogen colonization and obtain a competitive advantage. The antimicrobial mechanisms of action differ among bacteriocins, yet common mechanisms are the elevation of membrane permeability and prevention of nucleic acid and/or cell wall protein synthesis.^175^ *Bacillus subtilis* 3, *Weissella confuse* PL9001, *Lactobacillus gasseri* Kx110A1, *Lactobacillus brevis* ATCC 14869, *Lactobacillus bulgaricus*, and *L. reuteri* ATCC 55730 demonstrated inhibitory activity against *H. pylori* through bacteriocin production.^167,173^ Less-studied antimicrobial compounds in probiotics are siderophores that prevent pathogen access to iron, biosurfactants that interrupt or lyse pathogen cell membrane, and adhesion inhibitors, which interfere with the pathogen adhesion to epithelial cells and consequently prevent its virulence function.^175^

### Immune promotion

Probiotic strains may indirectly suppress *H. pylori* infection through the host immune response promotion by stimulating the activity of phagocytoses and natural killer (NK) cells, modifying phenotype and cytokine pattern of DCs, as well as increasing antibody and anti-inflammatory cytokines secretion.^176^ Interestingly, researchers reported that viable and non-viable bacteria had a different impact on the host cellular gene expression, suggesting the importance of both microbial cell surface and actively released substances on the gut transcriptome.^177^

*B. infantis* 35624 and *B. breve* YIT10347 activate the intestinal DCs by interacting with TLRs and stimulating RA metabolism. As a result, DCs activation elevates the expression of IL-10 and the number of Foxp3^+^ Treg and type 1 regulatory T (Tr1) cells. Moreover, *L. rhamnosus* GG and *L. acidophilus* can reduce the number of Th17 cells and the expression of IL-23 and IL-17 cytokines through prevention of STAT3 and NF-κB signaling and further shift the balance between pro-inflammatory M1 and immunosuppressive M2 macrophage toward M2 phenotype.^178^ In contrast, *Bifidobacterium animalis* spp. *lactis* Bl 5764 is able to promote IL-17A expression by CD4^+^ T lymphocytes in vitro. *L. reuteri* Lr 5454 co-culture with DCs can promote Tregs, and regenerating islet-derived protein 3-beta (Reg3b) expression in a NOD2-dependent manner and further induce IL-22 production.^179^ IL-22 plays an imperative role in preserving gut homeostasis and tissue regeneration. Furthermore, this cytokine accelerates the colonization of *Phascolarctobacterium* bacterium and thereby prevents *C. difficile* infection (CDI).^180^

Immunoglobulin A (IgA) as the main immunoglobulin isotype in the gut mucosa, regulates bacterial translocation and interferes with bacterial toxicity.^181^ *L. acidophilus* and *B. longum* are the major probiotic species demonstrated to increase IgA production from B cells in the intestinal lamina propia.^182^ Intestinal secretory IgA antibodies coat bacteria to prevent them from adhering the epithelium and barricading inflammation development. However, in vitro studies indicated that commensal microorganisms coated with IgA can grow without remarkable alteration. Moreover, high-affinity IgA coating elevates the risk of bacterial invasion and activation of inflammatory pathways. As *H. pylori* expresses receptors detecting IgA glycoprotein motifs, IgA attachment to these surface receptors improves *H. pylori* adhesion to the epithelial layer and facilitates its colonization.^183^

## Probiotic supplementation and gut microbiota alteration

Regarding *H. pylori* eradication, multiple studies investigated the impact of probiotic administration on the gut microbiota composition (Table 1). In the following sections, we aim to discuss the bioactivity of microbiota that noted significantly altered within the gastrointestinal tract of individuals who underwent *H. pylori* eradication by probiotic supplementation.

**Table 1.** Summary of studies examining the effects of probiotic co-supplementation to *H.* *pylori* eradication on the human gut microbiota.

Studies	Design	Eradication therapy	Probiotic strain/placebo	Methodology	Post-therapy evaluation (week after baseline)	Group specific alteration in the gut microbiota	
						Antibiotic/placebo group	Probiotic group
Oh et al., 2016^184^	10 subjects in each group	Amoxicillin 1 g bid, clarithromycin 500 mg bid, lansoprazole 30 mg bid, 14 days	*Streptococcus faecium* 9 × 10^8^, *Bacillus subtilis* 1 × 10^8^, twice daily, 14 days	16S rRNA gene-pyrosequencing (V1-V3)	2 weeks	*Citrobacter, Klebsiella, Pseudomonas*, and *Escherichia*	NS
Oh et al., 2016^185^	Age: 44–55 3 subjects in each group	Amoxicillin 1 g bid, clarithromycin 500 mg bid, lansoprazole 30 mg bid, 14 days	*Streptococcus faecium* 9 × 10^8^, *Bacillus subtilis* 1 × 10^8^, twice daily, 14 days	Whole metagenome sequencing. Miseq platform (Illumina)	2 weeks	*Klebsiella pneumoniae, Prevotella stercorea* ↑	*Lactobacillus ruminis, Escherichia coli, Bacteroides coprocola* ↑
Chen et al., 2018^99^	Age: 18–70 Antibiotic group: 32 subjects Probiotic group: 31 subjects	Amoxicillin 1 g bid, colloidal bismuth pectin 400 mg bid, furazolidone 100 mg bid, pantoprazole 40 mg bid, 14 days	*Clostridium butyricum* CBM 588 40 mg tid, 14 days	16S rRNA gene (V3-V4). Miseq platform (Illumina)	2 weeks	Lentisphaerae ↓ Proteobacteria, Cyanobacteria ↑ *Lactococcus raffinolactis, Lactobacillus sakei, Acinetobacter baumannii* NIPH60 ↑	Fusobacteria, Tenericutes ↓ Actinobacteria, Cyanobacteria ↑
					8 weeks	NS	NS
Wu et al., 2019^186^	Age: 18–65 20 subjects with dudenal ulcer in each group	Amoxicillin 1 g bid, clarithromycin 500 mg bid, esomeprazole 20 mg bid, 14 days	*Bacillus subtilis* and *Enterococcus faecium* coated capsules 500 mg tid, 6 weeks following eradication	16S rDNA (V4)	2 weeks	*Dialister*	*Actinomadura, Atopobium, Brevundimonas, Butyrivibrio, Coprococcus, Coraliomargarita, Corynebacterium, Desulfobacca, Desulfobulbus, Eggerthella, Faecalibacterium, GOUTA19, Helicobacter, Lewinella, Oscillospira, Rhodobacter, Rhodoplanes, Roseburia, Roseomonas, Rubrivivax, Thauera, Thiobacillus*
					6 weeks	*Dialister, Plesiomonas*	*Actinomadura, Anaerofilum, Candidatus Nitrososphaera, Candidatus Solibacter, Coprococcus, Coraliomargarita, Dechloromonas, Desulfomonile, Desulfobulbus, Dok59, Dorea, Leuconostoc, Luteimonas, Lewinella, Luteolibacter, Neisseria, Nitrosopumilus, Oscillospira, Parapedobacter, Planctomyces, Thauera, Syntrophobacter, Syntrophomonas, T78, Thermomonas, Thiobacillus*
					10 weeks	*Dialister*	*Achromobacter, Actinomyces, Cupriavidus, Bradyrhizobium, Candidatus Solibacter, Coprococcus, Chelativorans, Cetobacterium, Thiobacillus, Leuconostoc*
Cárdenas et al., 2020^187^	Age: 18–55 Antibiotic group: 22 Probiotic group:16	Amoxicillin 1 g tid, tinidazole 1 g qd, omeprazole 40 mg bid, 14 days	*Saccharomyces boulardii* CNCM I-745 750 mg qd, 14 days	16S rRNA. Miseq platform (Illumina)	2 weeks	NS	*Prevotella, Lachnospira, Ruminococcus* ↓ *Gammaproteobacteria* ↑
					6 weeks	NS	*Prevotella, Lachnospira, Ruminococcus* ↓ *Gammaproteobacteria* ↑
Kakiuchi et al., 2020^188^	Antibiotic group: 26 Probiotic group: 23	Amoxicillin 750 mg bid, clarithromycin 400 mg bid, vonoprazan 20 mg bid, 7 days	BFR tid, 7 days	16S rDNA (V3-V4). Miseq platform (Illumina)	1 week	*Collinsella, Bifidobacterium* ↓	*Blautia* ↑
Tang et al., 2020^189^	Age: 18–65 Placebo group: 74 Probiotic group: 77	Amoxicillin 1 g bid, bismuth potassium citrate 220 mg bid, furazolidone 100 mg bid, esomeprazole 20 mg bid, 14 days	*Enterococcus faecium* 4.5 × 10^8^ and *Bacillus subtilis* 5.0 × 10^7^, or maltodextrin tid, 4 weeks	16S rRNA (V3-V4). Miseq platform (Illumina)	2 weeks	*Dialister, Anaerotruncus, Megasphaera*	*Oscillospira, Citrobacter, Enterococcus*
					4 weeks	*Collinsella, Sutterella, Ruminococcus*	*Thermomonas, Bacillus, Lactobacillales, Enterococcus*
					6 weeks	*Coprococcus*	*Bulleidia, Anaerofustis*
					8 weeks	*Faecalibacterium*	*Phascolarctobacterium*
Guillemard et al., 2021^190^	Age: 18–65 68 subjects in each group	Amoxicillin 1 g bid, clarithromycin 500 mg bid, pantoprazole 40 mg bid, 14 days	*Lacticaseibacillus paracasei* CNCM I-1518, *Lacticaseibacillus paracasei* CNCM I-3689, *Lacticaseibacillus rhamnosus* CNCM I-3690, *Streptococcus thermophilus* (CNCM I-2773, CNCM I-2835, CNCM I-2778), *Lactobacillus delbrueckii* subsp *bulgaricus* CNCM I-2787, 28 days	16S rRNA (V3-V4)	2 weeks	*Enterorhabdus, Escherichia-Shigella, Roseburia, Leuconostoc, Akkermansia*	*Parasutterella, Howardella, Slackia, Desulfovibrio, Streptococcus, Lactobacillus, Alloprevotella, Succiniclasticum*
					4 weeks	*Enterorhabdus, Escherichia-Shigella, Roseburia, Megaphaera*	*Lactobacillus, Streptococcus, Butyricimonas, Methanobrevibacter, Pediococcus, Parasutterella, Howardella, Slackia, Desulfovibrio*
					6 weeks	*Enterorhabdus, Escherichia-Shigella, Roseburia, Megaphaera, Klebsiella, Succiniclasticum*	*Pediococcus, Parasutterella, Howardella, Slackia, Desulfovibrio, Megamonas, Lachnospira, Catenibacterium, Butyricimonas*
Yang et al., 2021^191^	Age: 18–70 94 subjects in each group	Amoxicillin 1 g bid, clarithromycin 500 mg bid, esomeprazole 20 mg bid, 14 days	Non-viable *Lactobacillus reuteri* DSM17648 2 × 10^10^ bid, 2 weeks pre-treatment	16S rRNA (V3-V4)	2 weeks	Proteobacteria *Escherichia-Shigella*	Bacteroidota *Bacteroides, Fusicatenibacter*
					8 weeks	NS	*Faecalibacterium, Subdoligranulum*
Yuan et al., 2021^192^	Age: 18–30 Antibiotic group: 34 Probiotic group: 31	Amoxicillin 1 g bid, clarithromycin 500 mg bid, potassium bismuth citrate 200 mg bid, esomeprazole 20 mg bid, 14 days	Bifidobacterium tetravaccine tablets included *B. infantis* >0.5 × 10^6^ CFU/tablet, *L. acidophilus* >0.5 × 10^6^ CFU/tablet, *E. faecalis* >0.5 × 10^6^ CFU/tablet, *B. cereus* >0.5 × 10^5^ CFU/tablet, three tablets each time, three times a day, 14 days	16S rRNA (V3-V4). Miseq platform (Illumina)	10 weeks	Gastric mucosa: *Erysipelatoclostridium, Ralstonia*	Gastric mucosa: *Lachnospiraceae* UCG 006
						Gastric juice: Proteobacteria was more abundnt than Firmicutes *Fusobacterium, Mycoplasma* (*Tenericutes), Leptotrichia, Campylobacter*	Gastric juice: Firmicutes was more abundnt than Proteobacteria *Ruminococcaceae* UCG014, *Eubacterium ventriosum*

qd, once a day; bid, twice a day; tid, three times a day; NS, not significant

### Single-strain probiotic supplementation

*C. butyricum* is an anaerobic bacterium that consumes undigested dietary fibers and mainly produces butyrate and acetate. Although some *C. butyricum* strains are equipped with toxins, others are antibiotic-sensitive and free of pathogenic markers and clostridial toxin genes.^193^ In particular, *C. butyricum* CBM 588 can inhibit gastrointestinal inflammation and side effects of antibiotic treatments, such as diarrhea. Consequently, oral administration of this probiotic might prevent inflammation-associated diseases such as UC.^194^ Chen et al. reported that *C. butyricum* CBM 588 co-supplementation with *H. pylori* quadruple therapy exhibited a significant reduction in Fusobacteria and Tenericutes phyla as well as an increase in Actinobacteria phylum following *H. pylori* eradication. However, *Lactococcus raffinolactis, Lactobacillus sakei*, and *Acinetobacter baumannii* NIPH60 were significantly increased only in the antibiotic group.^99^

Disregarding health conditions, over 100 uncultured Tenericutes have been recently discovered in the human gastrointestinal metagenome. Although the complex behavior of this phylum is not fully understood, Tenericutes bacteria in the host gastrointestinal tract demonstrated a substantial reduction in their genomes and metabolic capacities compared to environmental Tenericutes.^195^ Furthermore, Tenericutes is suggested to play a key role in the host metabolic pathways, such as bile acid metabolism.^196^ However, pathogenic species of this phylum are presented with virulence factors including hydrogen peroxide, toxins, surface polysaccharides, and sialic acid catabolism.^195^ Therefore, the reduction in the population of this taxon may cause various metabolic changes in the host, which needs further in-depth investigations at the strain level. Moreover, Fusobacteria is not prevalent nor relatively enriched in non-colorectal cancer individuals.^197^ This genus can stimulate cancer cell survival through modulation of STAT3, janus kinase 1 (JAK1), and MYC oncogenes and further induce tumor cell invasion by promoting IL-8 expression.^198^ Consequently, Fusobacteria depletion in the probiotic supplemented group may indicate a potentially beneficial effect for *C. butyricum* CBM 588 consumption during *H. pylori* eradication. On the other hand, *A. baumannii* bacteria are opportunistic pathogens and mainly contribute to ventilator-associated pneumonia and bloodstream infections.^199^ This pathogen has become a global health-care problem owing to the several mechanisms underlying its antibiotic resistance.^200^ As a result, the enrichment of this pathogenic species in the antibiotic group is consistent with the foregoing favorable effectiveness of probiotic administration. However, *L. sakei* that enriched in the antibiotic group is beneficially involved in obesity, cardiovascular disease, and gastrointestinal inflammation.^201^

*Enterococcus faecium* strains are particularly adaptive to their respective environment owing to their salt and acid tolerance. Although *E. faecium* are antibiotic-resistant infectious agents, they are hardly reported to induce infection in the human body. Moreover, certain *E. faecium* and *E. faecalis* strains are the only enterococci bacteria supplemented as probiotics.^202^ Biofermin-R (multidrug-resistant preparation of *E. faecium* 129 BIO 3B-R) administration with *H. pylori* triple therapy demonstrated beneficial advantages.^188^ This probiotic strain was reported to promote *Blautia* genus colonization, which is most commonly accompanied by probiotic activities.^203^ The reduced proportion of *Bifidobacterium* genus in the antibiotic treated group further highlights the delicate impact of Biofermin-R supplementation on preserving the abundance of probiotic genera among gut bacteria.

### Multi-strain probiotic supplementation

A randomized, controlled trial conducted in Germany demonstrated the advantage of probiotic co-supplementation in *H. pylori* eradication.^190^ In this study, the intestine of probiotic supplemented individuals was the residence of a higher proportion of *Slackia* bacteria that are suggested beneficially involved in the host isoflavone, fat, and energy metabolism.^204,205^ On the other hand, *Fusobacterium* that was enriched in the antibiotic group was correlated to digestive disorders, gastrointestinal inflammation, and colorectal carcinoma.^206,207^ However, *Desulfovibrio*, as Gram-negative sulfate-reducing bacteria, produce hydrogen sulfide and lipopolysaccharide and might contribute to the pathogenesis of Parkinson’s disease;^208^ consequently, the increased proportion of these bacteria in the gut bacterial community may cause post-therapy adverse effects following probiotic consumption. Moreover, *Methanobrevibacter* that enriched in the probiotic group are reported more abundant in Parkinson’s disease and gut dysbiosis.^209^ On the other hand, *Roseburia*, as major butyrate-producing bacteria in the intestine, can reduce oxidative stress, repair intestinal mucosa, and suppress intestinal inflammation.^210^ Therefore, the increased abundance of *Roseburia* bacteria in the antibiotic group may accelerate gut rebiosis after *H. pylori* treatment.

*B. subtilis* bacteria are consists of mesophilic, neutrophilic, and some pH tolerant strains with the capacity to produce a vast diversity of antimicrobial compounds.^211^ Several studies used *B. subtilis* and *E. faecium* combination as oral supplemented probiotic and further evaluated their synergic effect on *H. pylori* eradication, such as the research conducted by Oh et al. exhibiting that resistant bacteria to clarithromycin and amoxicillin, including *Citrobacter, Klebsiella, Pseudomonas*, and *Escherichia*, were significantly enriched in the antibiotic group than probiotic-supplemented group.^184^ *Klebsiella pneumoniae*, known as Gram-negative opportunistic pathogens, are responsible for the respiratory tract, urinary tract, and bloodstream infections. Due to the antibiotic resistance and hypervirulent characteristic of *Klebsiella pneumoniae* strains, clinical management of this pathogen has become progressively challenging.^212^ Oh et al. further reported the increased abundance of *Prevotella stercorea* in the antibiotic group, whereas *Lactobacillus ruminis* were enriched in the probiotic group.^185^ *P. stercorea* has been suggested to be positively correlated with the expression of mucosal pro-inflammatory cytokines especially TNF-α.^213^ Despite the poorly understood interaction of *L. ruminis* with the host biofunction, these bacteria may stimulate immune response through TLR2-mediated NF-κB activation and inhibit the growth of pathogens by acid secretion and competition for binding sites.^214^

As a result of probiotic administration for *H. pylori* treatment, Tang et al. reported the enrichment of beneficial bacteria including *Oscillospira*, Lactobacillales, and *Phascolarctobacterium* in the feces of probiotic-supplemented individuals.^189^ Although some studies indicated a positive correlation between *Oscillospira* and intestinal inflammation, it has been demonstrated that the relative abundance of *Oscillospira* is negatively associated with the expression of pro-inflammatory MCP-1, as well as the development of UC, IBD, and pediatric nonalcoholic fatty liver disease (NAFLD); therefore, *Oscillospira* is a candidate for the next-generation probiotics.^215^ Moreover, Lactobacillales of the Bacilli family can stimulate the innate and adaptive immune system and suppress inflammation by regulating IL-17 production.^216^ *Phascolarctobacterium* are reduced in hepatitis B virus-infected patients^217^ as well as individuals with postpartum depression disorder.^218^ As succinate consumers, *Phascolarctobacterium* bacteria can interfere with the colonization of succinate-consuming bacteria; therefore, preventing CDI.^219^ On the other hand, pathogenic bacteria have been reported to be enriched in the antibiotic group as *Dialister, Sutterella*, and *Collinsella* are mainly responsible for gut inflammation, liver diseases, and digestive disorder.^220–222^ Furthermore, *Anaerotruncus* that enriched in the antibiotic group are butyrate-producing bacteria with a positive correlation to saturated fatty acid and cholesterol intake; therefore, they are involved in obesity and NAFLD-associated hepatocellular carcinoma (HCC).^223,224^ *Citrobacter* genus presented a low virulence activity following their colonization in the gastrointestinal tract. However, increased abundance of *Citrobacter* species might lead to severe diseases in respiratory and urinary tract, central nervous system, bloodstream, and intestines in the probiotic supplemented patients.^225^ *Anaerofustis* genus is associated with movement and psychiatric disorders as well as pro-inflammatory activities.^226^ Furthermore, decreased starch degradation, possibly as a result of *Collinsella* reduction, leads to low levels of SCFAs production and weakens the gut epithelial barrier and host immune response in the probiotic group.^227^ Moreover, certain commensal bacteria including *Megasphaera, Ruminococcus*, and *Coprococcus* were significantly increased in the antibiotic group. Although some studies indicated that *Ruminococcus* species, particularly *Ruminococcus gnavus*, are correlated with T2D, CD, and UC,^228,229^ certain species such as *Ruminococcus bromii*, are abundant in healthy individuals and may lower cardiovascular risk and provide anti-inflammatory compounds through carbohydrate degradation.^230,231^ Furthermore, the proportion of gut *Ruminococcus* species is possibly associated with the number of CD8^+^ Treg cells in the human body, and thereby *Ruminococcus* bacteria may lower the risk for developing type 1 diabetes (T1D).^232^ *Megasphaera* are capable of SCFAs synthesis, osmotic diarrhea regulation, and host immune response promotion.^233^ Moreover, *Coprococcus* are inversely correlated to depression, lung cancer, and Parkinson’s disease.^234–236^

In consistent with the aforementioned studies, Wu et al. reported *Dialister* and *Plesiomonas* as the main genera in the patients undergoing *H. pylori* triple therapy regimen,^186,187^in which *Plesiomonas shigelloides*, as a single species in the *Plesiomonas* genus, is involved in gastrointestinal disorders including gastroenteritis and diarrhea.^237^ Nevertheless, some pathogenic bacteria, such as *Achromobacter, Actinomyces*, and *Cupriavidus*, were enriched during the study follow-up of the probiotic-supplemented group.

### Non-viable probiotic supplementation

The capacity of supplemented probiotics to temporarily or persistently colonize the gut mucosa and whether it is essential for their effects on the host biofunction are yet to be fully elucidated. A vast majority of researchers examined the successful probiotic colonization in the host mucosal layer by the proportion of probiotic bacteria in stool without direct assessment of mucosal samples.^238^ In a recent study, the comparison of fecal and mucosal expansion of supplemented probiotic species demonstrated that fecal presence of probiotic strains cannot identify permissive and resistant individuals, suggesting the passage of probiotic bacteria through the gastrointestinal tract without substantial adhesion nor colonization.^239^ Consequently, some studies investigated the effects of probiotic strains without the colonization capacity through the administration of dead and inactivated microorganisms, also termed as paraprobiotics.

Through leveraging a non-viable probiotic to reduce the cost and biological risk of treatment, Yang et al. demonstrated *Fusicatenibacter, Bacteroides, Faecalibacterium*, and *Subdoligranulum* as the main genera in the stool sample of individuals undergoing *H. pylori* triple therapy plus probiotic regimen.^191^ Several *Bacteroides* species are commensal bacteria providing nutrition and vitamins and playing a key role in cancer immunotherapy and prevention.^240^ *Faecalibacterium* genus mainly promote the host immune system by producing anti-inflammatory substances such as butyric acid and bioactive peptides; thereby, the reduced proportion of *Faecalibacterium* bacteria is correlated with the progression of IBD.^241^ Although the exact bioactivity of *Subdoligranulum* are not fully understood, this genus is suggested to have probiotic properties, particularly in the host metabolic health.^242^ Moreover, *Fusicatenibacter* are involved in butyric acid production and inversely correlated with IL-8 expression.^243^ On the other hand, *Escherichia-Shigella*, as the abundant bacteria in the antibiotic group, are associated with macrophage cell death, gut inflammation, and diarrhea.^244,245^

A recent study conducted in China reported the advantage of multi-strain probiotic administration in which detrimental bacteria were enriched in the antibiotic group while commensal bacteria were more abundant in the probiotic group.^192^ *Lachnospiraceae* UCG 006 and *Eubacterium ventriosum*, as commensal bacteria, can protect the human intestinal against colorectal cancer by producing SCFAs.^246,247^ Furthermore, *Ruminococcaceae* bacteria are one of the main butyrate producers in the human digestive tract; therefore, promoting the integrity of the gut barrier.^248^ On the other hand, the increased proportion of *Leptotrichia* is a risk factor for colorectal cancer;^249^ however, certain *Leptotrichia* species might be inversely correlated to pancreatic cancer.^250^ Moreover, *Leptotrichia* is reported as an oral health-related genus, substantially enriched in healthy individuals without dental caries experience.^251^

As one of the major causes of gastroenteritis, *Campylobacter* genus prevalence increased during the last decade globally. Well-studied species within the *Campylobacter* genus are *C. jejuni* and *C. fetus*, mainly responsible for the vast majority of reported *Campylobacter* infections and bloodstream infections, respectively.^252^ Therefore, the enhanced colonization of *Campylobacter* bacteria in the antibiotic group may further emphasize the beneficial effect of paraprobiotic consumption. Although *Erysipelatoclostridium* are SCFAs producers, the relative abundance of this genus is demonstrated to be enriched in the intestine of patients with gout, metabolic syndrome, and IBS.^253,254^ Furthermore, *Ralstonia* is a genus of Gram-negative opportunistic bacteria causing infection in immunocompromised hosts.^255^ However, these bacteria are more abundant in *H. pylori*-negative individuals than infected patients.^256^ Consequently, *Erysipelatoclostridium* and *Ralstonia* enrichment may increase the risk of developing gastrointestinal inflammation and immune disorders in the antibiotic group.

## The pros and cons of probiotic supplementation

While the safety of probiotic strains constitutes decades of ongoing conflict, researchers have generally reported beneficial advantages of probiotic supplementation in maintaining the host indigenous microbiome and reducing drug-related adverse effects.^257^ Notwithstanding, probiotic-induced adverse effects are poorly investigated and less noted in clinical trials.^258^ It has been recently indicated that the exposure of neonates to probiotic species is associated with a higher risk of oral, respiratory, and gastrointestinal infection throughout the lifespan.^259^ Furthermore, probiotic co-supplemented therapies, especially with *Lactobacillus* species, might be correlated with a delayed and incomplete post-antibiotic recovery of normal host–microbiome balance resulting in a long-term gut dysbiosis.^260^

Although fecal microbial composition may not exactly indicate the intestinal mucosa-adherent microbiome,^261^ only a limited number of clinical trials have investigated the influence of probiotic consumption on the gastrointestinal microbiota in situ. Thus, till the performance of more accurate studies, comparing the microbiota profile of patient’s stool sample following probiotic interventions and various other conditions may roughly represent the long-term safety and efficacy of probiotic supplementation. In this regard, *C. butyricum* CBM 588 co-supplementation^99^ may inhibit the replication of commensal bacteria and cause metabolic disorder, meanwhile reducing the risk of colorectal cancer and preventing the overgrowth of certain opportunistic pathogens. Furthermore, consumption of single-strain probiotic Biofermin-R^188^ may potentially promote the colonization of commensal bacteria.

Concerning multi-strain probiotic supplementation, results from the study by Guillemard et al.^190^ indicate the possibility of developing Parkinson’s disease and depletion of key butyrate-producing bacteria. However, this study may further point out the beneficial effect of probiotic supplementation through regulating the host isoflavone, fat, and energy metabolism, and reducing the risk of developing digestive disorders, gastrointestinal inflammation, and colorectal carcinoma. On the other hand, *B. subtilis* and *E. faecium* administration^184,185^ can inhibit the colonization of some opportunistic pathogens and potentially prevent respiratory tract, urinary tract, and bloodstream infections, as well as gastrointestinal inflammation. Gastrointestinal microbiota alteration following probiotic supplementation in the Tang et al.^189^ study further indicates the capacity of probiotic strains to prevent intestinal inflammation, as well as the development of UC, IBD, NAFLD, and CDI. However, the persistence of pathogenic genera in the probiotic supplemented group may provoke the emergence of severe gastrointestinal and extra-gastrointestinal diseases. Moreover, Wu et al.^186^ reported the enrichment of pathogenic bacteria in both the probiotic and the antibiotic groups. This might indicate limited effectiveness for the consumed probiotic strains in modulating drug-related adverse events.

Data from the study by Yang et al.^191^ may demonstrate the potential capacity of paraprobiotic consumption in promoting the host immune response, preventing intestinal inflammation and IBD development, and improving nutritional availability. Likewise, paraprobiotic administration^192^ may also promote the replication of SCFA-producing bacteria and prevent colorectal cancer, and preserve the integrity of the gut barrier. This might further inhibit pathogen colonization and lower the risk of developing bloodstream infection, metabolic syndrome, and IBS.

## Limitations and outstanding questions

Extensive complexity might describe the foremost characteristic of the host-microbiota multifaceted interplay. The individual gut microbiota composition at deep resolution levels and enormous structural diversity affecting bacterial functionality remain the challenge of grasping a profound knowledge in the field of host health and probiotic supplementation. This complexity leads to major limitations in determining the source of metabolites as host, probiotic, or indigenous microbiota;^54^ understanding the whole spectrum of condition-dependent and dose-dependent influence of probiotics; and exploring probiotic-host interaction in cell-culture systems or animal models.^131^ These limitations are frequently intermingled in ways that force conceptual and statistical interpretation toward substantial challenges. Nonetheless, several cohort studies with probiotic-oriented approaches guided experimental reductionism to elucidate mechanistic comprehension regarding probiotic involvement in human health and disease. Furthermore, the introduction of specific microbiome including probiotic strains into organoids by microinjection is a novel strategy to investigate the accurate cause and effect interaction between the host and microbiome.^262^

Recent advances in the knowledge about microbiota and the presentation of innovative experimental techniques would enable the integration of strain-specific features of probiotics and consideration of biologically related notions to accelerate the development of tailored therapeutics. The clinically controlled trials are the most practical way toward probiotic strain selection, in which a mechanism-oriented strategy should be pursued and certain questions should be contemplated. Is the probiotic interaction with the host mediated through the secretion of metabolites and alteration of the gut microbiota or colonization of the intestinal surface or other possible contact-dependent interactions? Should a next-generation probiotic strain be considered safe to provide a medical advantage in therapeutic interventions? What are the long-term consequences of probiotic-mediated alteration of the host microbiome? These critically important questions might be resolved by the novel paradigms of microbiome-on-a-chip technology, which can provide the real-time assessment of the host-microbiome interaction and explore the emergence of microbiome-related therapeutics.^263^

## Conclusions and outlook

The oral supplementation of a narrow diversity of Gram-positive bacteria demonstrated distinct amelioration in *H. pylori*-related clinical symptoms; however, the identified alteration in the gut microbiota demonstrates the possibility of intestinal or extra-gastrointestinal disease development later in life. Considering the enriched and depleted genera in the stool samples of probiotic-supplemented individuals, oral administration of multi-strain probiotics and paraprobiotics than single-strain probiotics might reduce the incidence of developing metabolic disorders. Yet, various characteristics of different strains in a common genus require innovative clinical approaches with high-throughput sequencing technology to determine gut microbiota alteration at the strain level.

Recent studies have expressed the intention of seeking next-generation probiotics and genetically modified microorganisms to promote the beneficial effect of probiotic supplementation in clinical outcomes. Concerning current probiotic strains, two main strategies are suggested for developing next-generation probiotics. One way is to identify the presence or absence of particular strains within the disease condition and investigate the efficiency of supplementing those strains to recover the health state. Another strategy is to harness a well-characterized probiotic strain to express a particular metabolites such as AMPs.^264^ Recent discoveries in biotechnology will accelerate the emergence of novel candidate probiotic strains and facilitate the transition from empiric into target-oriented interventions. Furthermore, the integration of nanotechnology with microencapsulation strategies may efficiently enhance the probiotic delivery system and thereby provide a regulatory framework to reduce the metabolic consequences of probiotic supplementation. Large-scale population studies with broad-spectrum antibiotic regimens and probiotic strains, as well as germ-free mice modeled by the human microbiome, will shed light on the long-term outcome of probiotic supplementation and elucidate unconventional ways to leverage diet and clinical interventions and personalize them to the subjects’ biology and microbiota.

## Data Availability

Data sharing is not applicable to this article as no new data were created or analyzed in this work.
